# Improvement in self-reported cognitive functioning but not in rumination following online working memory training in a two-year follow-up study of remitted major depressive disorder

**DOI:** 10.3389/fpsyt.2023.1163073

**Published:** 2023-06-05

**Authors:** Eivind Haga Ronold, Sunniva Brurok Myklebost, Åsa Hammar

**Affiliations:** ^1^Department of Biological and Medical Psychology, University of Bergen, Bergen, Norway; ^2^Division of Psychiatry, Haukeland University Hospital, University of Bergen, Bergen, Norway; ^3^Department of Clinical Sciences Lund, Psychiatry, Faculty of Medicine Lund University, Lund, Sweden; ^4^Office for Psychiatry and Habilitation, Psychiatry Research Skåne, Skåne, Sweden

**Keywords:** remission, digital interventions, longitudinal study, relapse prevention intervention, computerized working memory training, self reported cognitive deficits

## Abstract

Self-reported subjective cognitive difficulties (subjective deficits) and rumination are central residual cognitive symptoms following major depressive disorder (MDD). These are risk factors for more a severe course of illness, and despite the considerable relapse risk of MDD, few interventions target the remitted phase, a high-risk period for developing new episodes. Online distribution of interventions could help close this gap. Computerized working memory training (CWMT) shows promising results, but findings are inconclusive regarding which symptoms improve following this intervention, and its long-term effects. This study reports results from a longitudinal open-label two-year follow-up pilot-study of self-reported cognitive residual symptoms following 25 sessions (40 min), five times a week of a digitally delivered CWMT intervention. Ten of 29 patients remitted from MDD completed two-year follow-up assessment. Significant large improvements in self-reported cognitive functioning on the behavior rating inventory of executive function-adult version appeared after two-years (*d* = 0.98), but no significant improvements were found in rumination (*d* < 0.308) measured by the ruminative responses scale. The former showed moderate non-significant associations to improvement in CWMT both post-intervention (*r =* 0.575) and at two-year follow-up (*r =* 0.308). Strengths in the study included a comprehensive intervention and long follow-up time. Limitations were small sample and no control group. No significant differences between completers and drop-outs were found, however, attrition effects cannot be ruled out and demand characteristics could influence findings. Results suggested lasting improvements in self-reported cognitive functioning following online CWMT. Controlled studies with larger samples should replicate these promising preliminary findings.

## Introduction

Major depressive disorder (MDD) is a leading burden of disease globally. This trend has been enhanced by the COVID-19 pandemic (1), and the burden is in large part due to the high rates of relapse seen in MDD (2). New episodes are linked with persisting residual symptoms following affective remission including subthreshold depressive symptoms, fatigue, sleep problems, rumination, and cognitive deficits (3), contributing to relapse or recurrence and a more severe courses of illness (4). A large meta-analysis found small to large effects sizes for cognitive deficits in remitted MDD populations, measured objectively by neuropsychological tests, across most domains of cognitive functioning, *excluding* auditory attention, general cognitive ability, autobiographical memory, in addition to inhibition unconstrained by speed (5). In addition, self-reported subjective cognitive difficulties (subjective deficits) are prevalent in acute states of MDD (6); and as residual cognitive symptoms following MDD (7, 8). Subjective deficits are of clinical relevance and are associated with lower quality of life and poorer functioning following MDD (9), and could contribute to the alarming rate of relapse seen in MDD (10, 11). Perhaps surprisingly, subjective deficits show small associations to cognitive deficits measured by objective experimental and clinical neuropsychological tests (5, 12) A meta-analysis investigating the correlation between subjective and objective measures of cognition found small significant associations between some measures, with most consistent effects between questionnaires measuring shifting and tests measuring shifting (13). Some studies suggest that subjective cognition show associations to symptoms of MDD rather than to neuropsychological tests (14, 15). In the research literature objective cognitive tests performed in a controlled environment is recommended as a measure of cognitive deficits in depression (16). Self-reported cognition could be considered complementary to the performance on objective cognitive tests and measure different aspects of ecologically important (dys)function (12, 17–19). Importantly, cognitive deficits are not fully remediated by traditional treatments (20, 21), and a few interventions target residual cognitive difficulties in the remitted phase of MDD, including medication, brain stimulation and cognitive remediation (22). Little is known, however, about the long-term effects of interventions targeting cognition following MDD, as was evident by a recent meta-analysis (23). There is also a lack of research reporting long-term outcomes in subjective cognitive deficits, with a few exceptions (24–28). Overall, the results from these studies reporting short- and long-term outcomes have been mixed Thus, exploring how new interventions influence subjective deficits over time is of importance.

Another important residual cognitive symptom following MDD is rumination. Rumination is an emotion regulation strategy were one passively brood over self-related negative experiences and is hypothesized to be related to deficits in working memory (WM) for negative material, sometimes termed *hot-cognition* (26, 29–31). Associations between objective cognitive tests and rumination are small, but significant associations have been established for WM (3), and subjective cognition (24). Rumination is a central risk factor following remission of mood symptoms (32), and predict relapse in remitted populations (4, 33, 34). Thus, deficits in cognition are suspected to be related to rumination, however, relationships to subjective deficits are unclear (24), and a recent review suggested that subjective deficits could cause rumination about not living up to one’s premorbid level, and thus increase relapse risk following MDD (19). Interventions improving cognition could thus reduce subjective deficits and rumination following MDD. Interventions like computerized working memory training (CWMT), could result in improvement of residual cognitive symptoms following depression (10, 23). CWMT has been shown to improve objective cognitive functions like executive functions and associations between rumination and WM for negative material (23, 35). Results are mixed regarding whether interventions like CWMT improves rumination however, with studies finding support for improvements in some residual symptoms like objective cognitive deficits, but not all, symptoms like subjective cognitive deficits, depressive symptoms, and some aspects of rumination (19, 36, 37). Even less is known about subjective deficits (19, 25, 26), and the lasting effects of interventions, with preliminary reports suggesting long term improvements in rumination and depressive symptoms, but not subjective deficits (25). Given the high relapse rate and incremental relapse risk in MDD, knowledge about the lasting effects of interventions targeting residual cognitive symptoms is vital.

Supplying and accessing interventions targeting residual symptoms in remission from MDD can be challenging, as there are limited resources such as trained therapists in health care systems to deliver targeted treatment, particularly in remission (4). One promising approach is to deliver pro-cognitive interventions digitally as this could increase access, agency, and reduce costs (38). A strength within the research field is that many pro-cognitive interventions are already delivered online or are computerized (23). However, digital delivery might have implications for motivation and engagement (39). It has therefore been suggested that that sufficient guidance by therapists can increase motivation and engagement (40). In sum, given limited studies in this area, there is a need to further investigate the long-term effects of on cost effective interventions on clinically relevant residual cognitive symptom like subjective deficits and rumination.

The current study investigated the long-term effects of a comprehensive digitally delivered CWMT intervention. If CWMT improves working memory (WM) control for negative material in hot-cognition, associated with rumination (35, 41), rumination would decline over time following CWMT. Ronold et al. (35) investigated CWMT effects on hot- and cold cognition, rumination, and depressive symptoms and found significantly improved effects on the two former, but not the latter. However, WM improvements could generalize to daily function, e.g., self-perceived cognitive function over time, and a study found associations between subjective deficits and later quality of life and psychosocial function (9), suggesting that long term development is important to investigate. Also improvements over time could reduce ruminative tendencies. It was thus hypothesized that CWMT would yield durable effects in self-reported cognitive function and rumination. The following research questions were investigated:

-How does self-reported cognitive function (subjective deficits) and rumination develop two-year following CWMT?
-Is CWMT improvement related to improvement in subjective deficits or rumination?

## Methods

### Participants and procedure

This study is based on two-year data from an open pilot study investigating the effects of CWMT. Participants in the study were assessed pre, post, one- and two-years after CWMT.

#### Recruitment, participant flow

Twenty-nine remitted participants assessed by the Montgomery Åsberg Depression Rating Scale (MADRS) (42) a score of ≤12, were included in the study initially [for more details see (35)]. Participants remained remitted at one and two-year follow up (MADRS score ≤ 12). One participant was missing from the completers group at 1 year follow-up. See Table 1 for demographical characteristics.

**Table 1:** Participant demographics, attrition and training outcomes.

Groups (males)	Completers *n* = 10 (4)	Drop out 2 years *n =* 9 (2)	Drop from intervention *n =* 10 (4)
	*M* (SD)	*M* (SD)	*M* (SD)
Age	36.5 (10.384)	38.3 (14.016)	33.56 (7.732)
Education	16 (2.11)	17.1 (1.45)	15.44 (1.74)
BRIEF pre	120.7 (25.18)	115.6 (20.18)	124.67 (25.23)
BRIEF post training*	110.5 (24.73)	116.44 (16.57)	***
BRIEF 1 year*	100.44 (18.44) *n =* 9	96 (23.895) *n* = 3	***
BRIEF 2 years	97.9 (19.04)	***	***
RRS pre	48.7 (14.637)	47.89 (13.743)	47.778 (14.636)
RRS post training*	47 (18.499)	45 (10.013)	***
RRS 1 year*	39 (12.32)	44.33 (13.65) *n* = 3	***
RRS 2 years*	41.3 (14.937)	***	***
% Improvement CWMT	53.3 (14.9)	48.7 (17.78)	***

Completers finished the intervention and came back for follow up assessments. Drop out from intervention did not complete the training. Drop out 2 years did not come back for follow up assessments in the years following pre-test. No significant differences were found between groups with small effect sizes, except for years of education with non-significant large effect size. BRIEF, behavior rating inventory of executive function (global executive composite score). RRS, ruminative responses scale. *p > 0.05 in improvement *** = not assessed due to drop out.

### CWMT intervention

A commercial CWMT program, Cogmed^™^ was used for cognitive training. The program consisted of several number-, letter-, and spatial span tasks with personalized, incremental difficulty. Participants worked approximately 40 min on different tasks each training day. The interventions were delivered *digitally*, online, were participants logged on through computer or laptop, lasted approximately 5 weeks, with roughly five days of training each week, and had weekly therapist guidance over telephone discussing progress and potential obstacles with the training, such as motivational issues. See Hammar et al. (10) for further description concerning the paradigm and follow-up.

### Ethics statement

All participants provided informed signed consent prior to participating in the respective studies. The study was conducted in accordance with the Helsinki declaration of Ethical Research regarding Ethical Principles for Medical Research Involving Human Subjects and the approved by the committee for ethical research in Western Norway (2014/1079). Participants were compensated through free access to the Cogmed^™^ platform and received a gift card (approximately 40$) at two-year follow up.

### Outcome-measures

#### Subjective cognitive function

Participants completed an inventory of self-report measure of cognitive difficulties in executive functions (behavior rating inventory of executive function-adult version; BRIEF-A) of which the 75 items global executive composite (GEC) was used to measure subjectively experienced cognitive difficulties. Raw scores were used to investigate changes, and high scores indicated poorer rating.

#### Rumination

Participants completed a 22-item self-report assessment of rumination, the Ruminative Responses Scale (RRS) to assess tendency for rumination in response to sad mood.

### Statistical analyses and improvement scores

**Figure 1:**
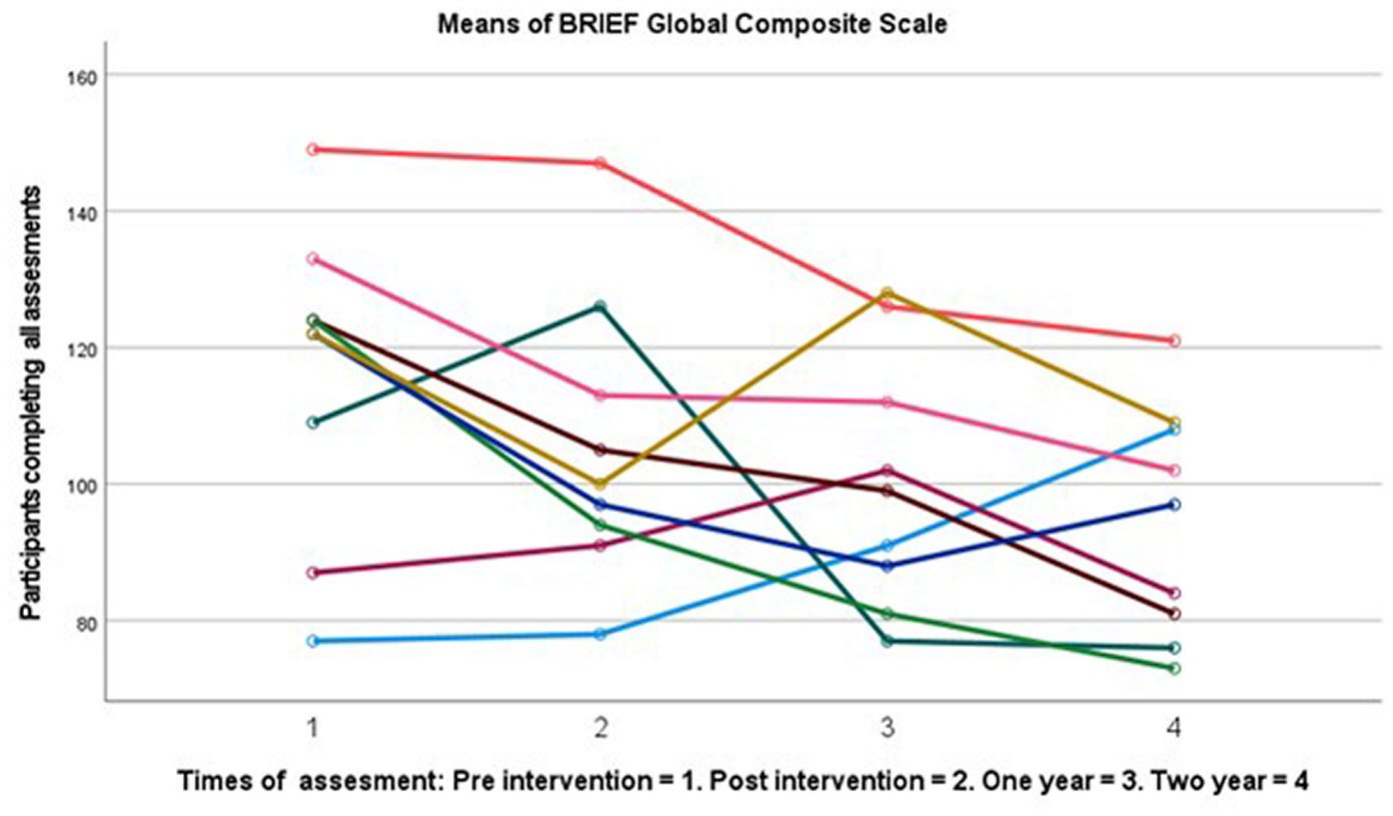
Spaghetti plot change in subjective cognition score over 2 years (*n* = 9).

**Figure 2:**
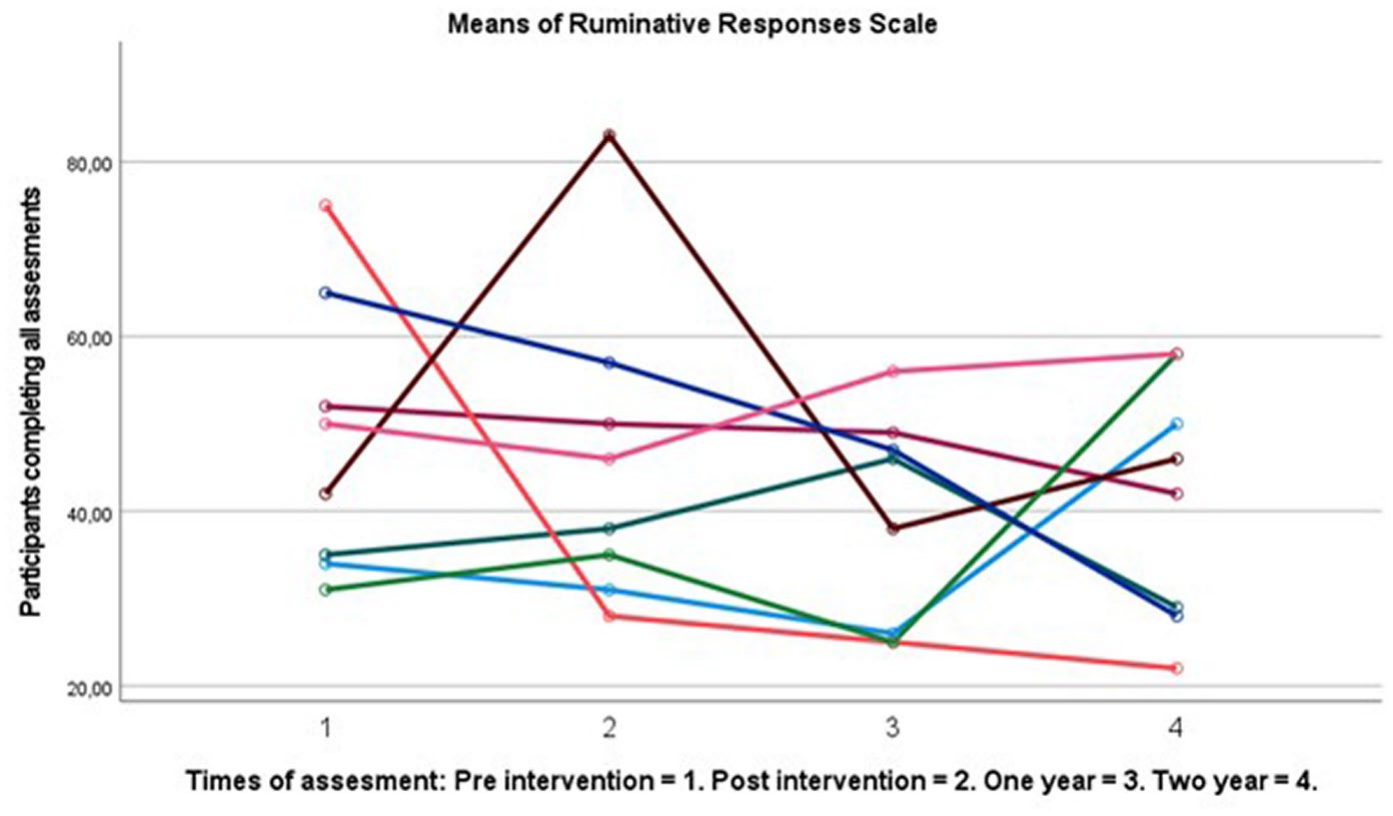
Spaghetti plot of change in RRS score over 2 years (*n* = 8).

All analyses were run in the Statistical Package for the Social Sciences (SPSS; version 28). Attrition was investigated through one way ANOVA. Repeated measures ANOVA investigated changes in subjective cognitive function and rumination from pre- to post intervention, 1 and two-year follow up. Paired sampled *t*-tests was used as follow up tests. Percent improvement in CWMT was calculated by the formula highest score—starting score/starting score * 100. Improvement scores were calculated by subtracting pre scores from post (immediately after the intervention)- and two-year follow up scores, so positive values represented improved scores, and bivariate correlations between CWMT and residual symptom improvement was calculated (Pearson’s *r*). Effect sizes was described as small, medium, and large according to Cohen (43). Because of the small sample spaghetti plots showing the individual trajectories in BRIEF (see Figure 1) and RRS (see Figure 2) were used to illustrate development of subjectively reported cognitive function and rumination.

## Results

There were no significant differences between completers and drop-outs on any of the clinical or demographic variables with small effects (see Table 1), suggesting limited attrition effects. Drop out was high at two-years follow up with 64%. Completers showed large significant improvements in subjective cognitive functioning *F* (3, 24) 3.676 *p* = 0.026, *η*^2^ = 0.315. Follow up tests showed a significantly changed BRIEF-A GEC score with large effect sizes from pre- (*M* = 120.7, SD = 25.17) to two-year post-intervention (*M* = 97.9, SD = 19.04), *t* (9) = 3.1, *p* = 0.013 *d* = 0.98. No other time point had significant improvements (see Table 1 for post-test and 1 year means). There were small and non-significant changes in rumination following the intervention at one- and two-year follow up. See Figures 1, 2 for a graphical representation of individual changes in subjective cognition and rumination. Correlation analyses suggested medium correlations between subjective cognition and CWMT improvement and subjective cognitive function both immediate and two-year following intervention, however these did not reach statistical significance *p* > 0.05 (see Table 2).

**Table 2:** Correlations between CWMT improvement and changes in rumination and subjective cognition.

	1.	2.	3.	4.	5.
1. CWMT improvement	1				
2. RRS pre-post change	−0.413	1			
3. BRIEF pre-post change	0.575*	−0.202	1		
4. RRS pre-2 years change	−0.062	0.649**	−0.088	1	
5. BRIEF pre-2 years change	0.308	−0.058	0.396	0.456	1

Pearson’s *r* values for completers (*n* = 10), medium associations appeared between CWMT improvement and improvement in subjective cognition from pre- to post intervention (assessment immediately after intervention) *approaching significance *p* = 0.083 (two-sided). **p < 0.05. BRIEF, behavior rating inventory of executive function (global executive composite change scores). RRS, ruminative responses scale change scores.

## Discussion

Long term improvements on self-reported cognitive symptoms following a digitally delivered CWMT intervention were found. Although the results must be considered preliminary and uncertain due to the small sample size and lack of control group, findings support the use of digitally delivered CWMT to improve subjective deficits. In fact, the improvements in subjective cognition were even larger compared to preliminary findings from long term follow up of *in-person interventions* cognitive training interventions (25). This could support better acceptability and feasibility of online tasks compared to other interventions (10, 26), especially for long term outcomes for subjective cognition.

Rumination did not improve; thus, interventions targeting emotional information processing and hot cognition might be more optimal. Hot cognition involves cognitive control for emotional material (29), and could be more related to depressive symptoms than classical tasks of cognitive and executive functions (44). Alternatively, the small sample was underpowered to detect changes in rumination. Hammar et al. (10), for instance, reported that a subgroup improving from CWMT showed decreased rumination. Several studies have suggested that subgroups with different cognitive profiles could exist in MDD (45, 46), and finding moderators for personalization and optimalization of cognitive training effects are important for future research (47). There are theoretical and empirical connections between WM and rumination (31), with several authors targeting this modality by cognitive training with various success (36). Thus, personalizing interventions and examine subgroups to identify those who show- and do not show benefits could be important (47). Alternatively, or in addition, rumination might show more connections to hot-cognition than cold-cognition (29, 35, 41, 44), and targeting “hot cognition” could be more effective in reducing rumination and affective residual symptoms of depression (48). Thus, future studies should investigate the ability of digitally delivered interventions to target more broadly, including hot cognition and rumination, and investigate the mechanisms of cognitive training on emotion regulation (37). The weekly therapist contact might have influenced results. However, since improvements increased even 1 year after the intervention, this could probably not fully explain results. The moderate association between improvement in self-reported subjective cognitive difficulties and the training paradigm could suggest specific effects of WM training on self-reported cognitive functions in daily life and might thus represent an example of far transfer effects, or at least near transfer across domains. Findings could be relevant to relapse prevention because of this, however more methodological rigorous controlled studies should investigate this further.

### Strengths, limitations, and suggestions for future research

This is the first study to report on the effects of digitally delivered cognitive enhancement interventions after two-years on residual cognitive symptoms. However, sample sizes were small-, dropout considerable, but comparable to other studies in the literature (23, 25). There were no differences on demographic or outcome variables between those completing the intervention and dropping out at pre intervention, however, attrition effects cannot be ruled out, and some participants might have relapsed during training and follow up. The study was underpowered to detect small changes. Thus, acceptability could be a challenge in the current study. Previous studies, however, suggested that the current CWMT paradigm is both acceptable and feasible to use (10, 49). No objective neuropsychological assessments of cognitive functions were reported, limiting generalizability to cognitive deficits on behavioral tasks. However, subjectively experienced cognitive difficulties could be of clinical relevance in the remitted phase, as it could be associated with risk of depression relapse and poorer outcomes following MDD (19). Another limitation concerning outcome measures is not including a functional transfer measure of cognitive effects, which is recommended to investigate cognitive interventions generalizability (50). Sample- and demand characteristics could partly explain results, and future studies should investigate factors related to drop out, include more participants, and implement designs controlling for these variables. In addition, the increasing improvements in subjective cognition over time suggested that it might take time for effects to generalize, and thus longitudinal studies of cognitive remediation with longer follow up time might be feasible. However, due to the high drop-out, longitudinal studies with digital follow up assessment might be more optimal for acceptability following CWMT interventions, and future longitudinal studies should plan for this.

## Conclusion

A group completing digitally delivered CWMT showed reduced levels of subjective cognitive deficits two-years following implementation. This shows significant promise in effective and available relapse prevention strategies. Thus, the digitally delivered working memory training shows promise in reducing subjective difficulties following MDD, and long-term effects of digitally delivered interventions could be comparable or supersede those seen in the limited research on interventions delivered in the clinic. However, given the methodological qualities of the current study, more controlled replication of results should be done. There were no significant improvements in rumination, underscoring the importance of continuing to develop interventions targeting important residual symptoms like hot cognitive processes for optimal relapse prevention.

## Data availability statement

The raw data supporting the conclusions of this article will be made available by the authors, without undue reservation.

## Ethics statement

The studies involving human participants were reviewed and approved by The committee for ethical research in Western Norway (2014/1079). The patients/participants provided their written informed consent to participate in this study.

## Author contributions

ÅH was PI for the study. ER and SM helped conduct the study. ER, SM, and ÅH formulated hypotheses and research questions. ER conducted analyses, wrote manuscript, constructed tables, and figures. ÅH and SM commented and edited the manuscript draft. All authors contributed to the article and approved the submitted version.

## Conflict of interest

The authors declare that the research was conducted in the absence of any commercial or financial relationships that could be construed as a potential conflict of interest.

## Publisher’s note

All claims expressed in this article are solely those of the authors and do not necessarily represent those of their affiliated organizations, or those of the publisher, the editors and the reviewers. Any product that may be evaluated in this article, or claim that may be made by its manufacturer, is not guaranteed or endorsed by the publisher.
